# Starting ambulance care professionals and critical incidents: a qualitative study on experiences, consequences and coping strategies

**DOI:** 10.1186/s12873-021-00500-9

**Published:** 2021-10-07

**Authors:** Jorik Loef, Lilian C. M. Vloet, Peter-Hans Vierhoven, Leonie van der Schans, Yvonne Neyman-Lubbers, Christine de Vries-de Winter, Remco H. A. Ebben

**Affiliations:** 1 Emergency Medical Service Brabant Midden-West-Noord, ‘s Hertogenbosch, The Netherlands; 2 Research Department of Emergency and Critical Care, HAN University of Applied Sciences, School of Health Studies, Nijmegen, the Netherlands; 3 IQ healthcare, Radboud University Medical Center, Radboud Institute for Health Sciences, Nijmegen, The Netherlands; 4 Dutch platform of Bachelor of Medical Health, Utrecht, The Netherlands; 5 Dutch Society of Bachelor of Medical Health, Utrecht, The Netherlands; 6 Emergency Medical Service Amsterdam, Amsterdam, The Netherlands

**Keywords:** Emergency medical services, Adaptation, Psychological, Trauma and stressor related disorders, Critical incidents

## Abstract

**Background:**

Ambulance care professionals are regularly confronted with critical incidents that increase risks for mental health disorders. To minimize these risks, it is important that ambulance care professionals adequately cope with critical incidents. Especially from the perspective of starting ambulance care professionals it is unknown which coping styles they use when experiencing a critical incident and how they are trained to cope with critical incidents. The aim of this study was to gain insight in (a) what starting ambulance care professionals describe as critical incidents, (b) how they experience these critical incidents and their consequences, (c) how they cope with these incidents, and (d) how they are trained and guided to cope with these incidents.

**Methods:**

A qualitative design with individual, semi-structured interviews was used. The data was analyzed by using inductive thematic analysis.

**Results:**

Twenty-two starting ambulance care professionals were interviewed of which, 11 were male. The age ranged from 23 to 31 years, with 11 participants being 27 years or younger. Three key-themes emerged that make an incident critical: (1) emotional connection versus emotional detachment, (2) feeling loss of control, and (3) incomprehension. All participants experienced several short to middle term physical, psychological and social consequences after encountering a critical incident. Starting ambulance care professionals applied different coping strategies during different phases of the ambulance care process: a mix of depersonification, focus on the medical task, support from colleagues and their own network, seeking confirmation, and distraction. Most starting ambulance care professionals don’t actively remember they received education about coping with critical incidents during their initial educational program. During and after traineeships, the workplace preceptor has a crucial role for starting ambulance care professionals to learn them how to cope with critical incidents.

**Conclusions:**

Three key-themes interact to make an incident more critical for starting ambulance care professionals. To cope with these critical incidents, starting ambulance care professionals use a variety of coping strategies. These results can be used to develop training and coaching for starting ambulance care professionals so they can adequately cope with critical incidents.

## Background

Ambulance care professionals are regularly confronted with potentially traumatizing events that may cause psychological distress, so called critical incidents. Literature describes several definitions of these critical incidents. Common characteristics are that it concerns an incident that overwhelms the ambulance care professional with unusually strong emotions, interferes with their ability to function or cope before, during or after the incident, and that is different from chronic workplace stressors [1–3].

When ambulance care professionals experience cumulative critical incidents, the risk for mental health disorders increases [4]. A recent systematic review on mental health disorders among ambulance personnel reported prevalence rates of 27% general psychological distress, 15% anxiety, 15% depression, and 11% Post Traumatic Stress Disorder (PTSD) [5]. Another study reported a 8.6% prevalence of burnouts amongst ambulance care professionals [6]. Compared to the general working population, the prevalence’s of these mental health outcomes are higher among ambulance care professionals [5, 6]. Compared to in-hospital care professionals, pre-hospital ambulance care professionals are more likely to screen positive for PTSD [7]. These mental health disorders affect ambulance care professional wellbeing by increasing the risk for sick leave and suicidal thoughts [8–10].

To minimize risks for malfunction and early retirement from ambulance care, it is important that ambulance care professionals adequately cope with critical incidents. Coping is defined as the behaviorally, cognitively and emotionally reaction to circumstances that require adaptation. A coping strategy is defined as the pattern of behavior that is predominantly employed when one faces a new or unusual situation [11].

Studies indicate that ambulance care professionals are frequently exposed to critical incidents, especially within 2 years after the beginning of their career [12, 13]. One of the assumptions in the ambulance care sector is that it is difficult for starting ambulance care professionals to cope with critical incidents. Especially from the perspective of starting ambulance care professionals it is unknown which coping styles they use when experiencing a critical incident and how they are trained to cope with critical incidents. Therefore, the aim of this study was to gain insight in (a) what starting ambulance care professionals describe as critical incidents, (b) how they experience these critical incidents and their consequences, (c) how they cope with these incidents, and (d) how they are trained and guided to cope with these incidents.

## Methods

### Design

We used a qualitative design and performed individual, semi-structured interviews. Qualitative approaches are suitable to gain insight from the perspective of participants, and are previously used in studies with comparable aims [2, 14, 15]. This study is reported in accordance with the COREQ statement [16]. The Ethical Research Committee of the HAN University of Applied Sciences critically appraised the research protocol and concluded that the study complied with the criteria of the Declaration of Helsinki on Ethical Principles for Medical Research Involving Human Subjects, applicable national laws (like the General Data Protection Regulation), and the Dutch code of conduct for Research Integrity (reference Ethical Research Committee HAN University of Applied Sciences: 263.04/21).

### Population and setting

In the Netherlands there are 25 emergency medical services (EMS) and there are routes in the nursing domain and a route in the medical domain to become qualified as ambulance care professional. The first, already existing route, is to become a registered nurse (RN), who follows several specialist educational programs, before entering a specific national EMS training course. In 2010 a second route was developed in the medical domain, the bachelor of health (BH). This route was developed when staff shortage in emergency care was developing in the Netherlands, and thus there was a need for personnel with a relative short training route with the same educational level. The BH route is a four-year educational program. This educational program consists of a 2 year common trunk, the last 2 years consist of a specialization in one of the following routes: emergency care (ambulance or ED), anesthesiology, operation room or cardiodiagnostic care. After graduation in the emergency care route, the BH follows a 9–12 month traineeship at the EMS to become registered as an ambulance care professional. In comparison to the already existing nursing route, the BH route takes approximately 5 years less to become qualified as ambulance care professional. When the BH enters the ambulance care setting, they are younger and less experienced in comparison to their colleagues from the nursing route, which emphasizes the need to gain insight in this specific group.

At the start of the study (April 2020) there were 29 registered BH working as ambulance care professional in 10 different EMSs. This relatively low number of 29 can be explained by the fact that students can choose different specialization routes during the BH program, and due to a lack of internships in ambulance care. Using total population sampling, all BH were invited to participate in the study via a personal email by two researchers (JL & RE). In this email the researchers introduced themselves and the study aim. Also, and information letter with information about the study protocol and informed consent was sent.

### Data collection

In April and May 2020, 4 trained students (2 BH students, 2 nursing students) under supervision of RE & JL conducted semi-structured interviews using a topic list. The topic list was based on literature on critical incidents [2, 3]. The topic list consisted of an explorative opening question on what makes an incident critical, followed by questions related to the main topics of this study: critical incidents, experiences and their consequences, coping, and training and guidance. The interviewer made sure that all topics were addressed, but no pre-structured questions besides the explorative opening question were formulated. Due to COVID-19 all 22 interviews were conducted through the online platforms Zoom and Skype. During each interview 2 interviewers and the participant were present. All interviews were recorded (audio and video), this provided the opportunity for the interviewers and supervisors to check data on relevance and saturation during an ongoing iterative process of data collection and analysis. Interviews lasted from 29 up to 74 min, with an average of 50 min.

### Data analysis

Data was analyzed using inductive thematic analysis, by identifying patterns and key-themes across all interviews without any pre-existing theoretical framework [17]. All interviews were transcribed verbatim. The second step was coding the transcripts using open and in-vivo codes (JL, RE). These codes were then grouped into key-themes and subthemes by two researchers (JL, RE.). To increase dependability and credibility, investigator-triangulation, peer reviews, and member checks were applied [18]. Investigator-triangulation was accomplished by involving two different researchers (supervisors) in the data-analysis phase. Re-analyses of raw data were made by a peer researcher. Finally, identified key-themes were sent to the participants for a member-check and supplementary comments. We used Microsoft Excel and Microsoft Word for qualitative analysis. All audio and video tapes, transcripts, informed consents and documents are stored at a secured working environment, to which only the supervisors have access.

## Results

### Participants

Twenty-two BH agreed to participate in the study, 6 BH did not respond, and 1 declined because of work pressure due to COVID-19. Of the 22 participants, 11 were male. The age ranged from 23 to 31 years, with 11 participants being 27 years or younger. 10 participants had 0–12 months of working experience after finishing their traineeship, 8 had 13–24 months of working experience, and 4 had 25–36 months of working experience.

**Fig. 1:**
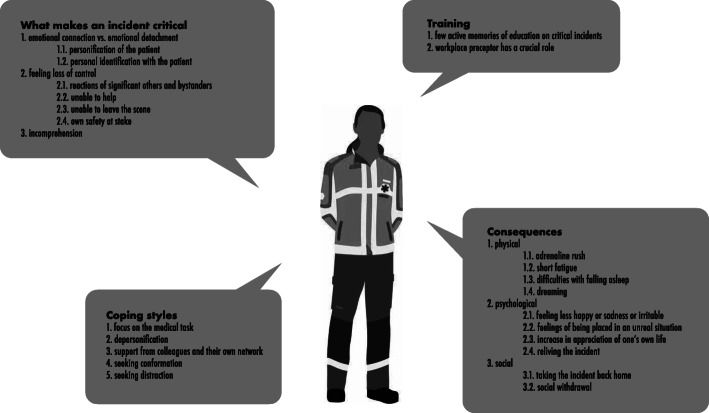
Results overview. Legend: Three key-themes that make an incident critical, the used coping styles before, during and after a critical incident, the consequences before and after dealing with a critical incident, and the training and guidance when dealing with a critical incident

Results for all aims are displayed in Fig. 1.

### Critical incidents: description and experience

From our analysis, three key-themes emerged that make and incident critical for starting ambulance care professionals: (1) emotional connection versus emotional detachment, (2) feeling loss of control, and (3) incomprehension. Critical incidents most mentioned by the participants are displayed in Table 1.

**Table 1:** Overview of most mentioned incidents in which the three key-themes made the incident more critical from the perspective of the ambulance care professional (alphabetical order)

• Aggression/violence/murder	
• Cases where one’s own safety is at stake	
• Domestic violence (abuse, neglect, harm)	
• Heavily traumatized/mutilated bodies	
• Planned ambulance runs where the patients tells his/her life story	
• Psychiatric patients	
• Resuscitations	
• Sick or dead children	
• Suicide	

#### Key-theme 1: emotional connection versus emotional detachment

Regarding ‘emotional connection versus emotional detachment’, all participants described this theme, and distinctioned two subthemes: personification of the patient, and personal identification with the patient.

#### Subtheme 1.1: personification of the patient

Participants described that they approach each patient as a medical case, and thereby they depersonify the patient. When the starting ambulance care professional gets to know more about the person behind the patient and the patient personifies, the incident can become more critical. For instance this happens when the starting ambulance care professional learns about the patients’ life and sees the patient in their personal context. The participants mentioned several ways to get to know the patient like: personal conversations, seeing pictures, reading letters and getting to know the patients’ significant others.

Some starting ambulance care professionals protect themselves from personification of the patient, by actively avoiding getting to know the patient when the event gets critical. These professionals do not look at the face of the patient, photos, and personal belongings.

*“I do not look the patient in the eyes, for instance during a resuscitation or when a patient has horrible injuries... the person becomes more real, that is something I try to prevent” (participant #18)*

*“Sometimes it is not the patient’s injury but the context surrounding the patient, for instance when family is present or when I get to know more about the person… The other time a young man died and I heard his girlfriend was pregnant and gave birth the same night, that was extremely emotional” (participant #6)*

*“Then, as we left for the hospice with the patient, his daughter held her new born baby in her arms. That moment was tough. It was not the fact that we saw a lot of blood or something, but it was all about being human” (participant #5)*

#### Subtheme 1.2: personal identification with the patient

All participants mentioned that when they can relate to the patient, and recognize themselves, relatives or their own personal living situations, they identify themselves with the patient and feel strong emotional connection. The personal identification that is mentioned is caused by a patient of the same age, a patient they personally know or a patient who lives in their own neighborhood. The main emotion felt within this personal identification by participants was fear. The starting ambulance care professionals feared that they or a relative or partner could become ill or injured as well.

*“If I recognize elements of the case or patient in my own life, I have increasing fear that it will happen to me” (participant #7)*

#### Key-theme 2: feeling loss of control

Regarding the second key-theme ‘feeling loss of control’ all participants mentioned that feeling loss of control made an incident more critical. Four subthemes were distinctioned: reactions of significant others and bystanders, unable to help, unable to leave the scene, and when their own safety is at stake.

#### Subtheme 2.1: reactions of significant others and bystanders

Most of the participants mentioned that reactions of involved significant others and bystanders contributed to an event being critical. The starting ambulance care professional is confronted with different types of behavioral and emotional reactions, from being quiet and cooperative, to (strong) emotions like crying or shouting. Participants responded differently to these reactions, and felt that they needed to work harder to keep control of the situation. Some felt in control while others felt a loss of control.

*“A case becomes more critical when there is a lot of family at the scene, who are interfering with the case and are very emotional” (participant #2)*

*“There were 150 people screaming and shouting which was impressive, but despite that the resuscitation continued” (participant #1)*

#### Subtheme 2.2: unable to help

All participants mentioned they used their pre-arrival preparation, patient assessment triangle at arrival, the ABCDE-method and their national protocol to structure the situation. They combine these methods and instruments with their own experience to feel secure and to become in control of the situation. Some starting ambulance care professionals perceived major incidents with interprofessional collaboration as chaotic and loss of control because they were not sure about their role and what to do. Others mentioned that being confronted with rare cases made them feel less in control. Some participants experienced psychiatric cases as difficult because they were unable to help and felt more loss of control.

*“When I am confronted with a case that I have done many times, I notice I feel more secure and in control of the situation and my emotions” (respondent #12)*

*“For me, those psychiatric cases are the most difficult...sometimes you are unable to help someone, it makes me feel helpless’ (respondent #7)*

#### Subtheme 2.3: unable to leave the scene

Some starting ambulance care professionals mentioned that when they were unable to leave the scene with the patient, this caused instant feelings of helplessness, and some reported feelings of panic.

*“When the condition of the patient is unstable and I want to leave the scene to transport the patient to the hospital...and I can’t leave because the patient is pinched...I felt helpless.. and I panicked” (participant #15)*

#### Subtheme 2.4: own safety at stake

Some starting ambulance care professionals mentioned that when their own safety was at stake, they felt loss of control over their own safety and thus the incident became more critical.

*“Sometimes you feel impregnable when wearing your ambulance outfit ... I am here to help right? And then all of a sudden people demand that you do things you don’t want to do, or people start to threaten you.” (participant # 20)*

#### Key-theme 3: incomprehension

Most participants mentioned cases where they experienced deep feelings of incomprehension, which makes an incident more critical. These cases could be divided into two groups. The first group consists of cases that happen to someone, like a sick child or a heavily traumatized body due to an accident or fall. Starting ambulance care professionals ask themselves existential questions like ‘why is this happening to a young child?’ or ‘is this normal?’. The second group consists of cases that are done to someone, like violence, abuse and neglect. Regarding these cases participants feel strong disbelief and could not find a deeper meaning in it.

*“This guy got stuck in the tree shredder and the device turned on and he disappeared within seconds. That is truly gruesome to see … That this can happen at all” (participant #16)*

*“There shouldn’t happen anything to children, that’s just not normal” (participant #14)*

*“What makes an incident critical for me is when I cannot relate to the perpetrator, I do not understand what he was thinking. Why does he do this to someone else? To cause so much sorrow (participant #17)*

### Experienced consequences of critical incidents

All participants experienced several short to middle term consequences after encountering a critical incident. These consequences could be divided into physical, psychological and social aspects. The consequences were experienced as more severe if there were multiple critical incidents within a short period of time. Also, almost all starting ambulance care professionals mentioned that their own personal health situation affects how they perceive the consequences and cope with the incident. Almost all participants expressed that they accept these consequences as part of their ambulance work, and did not experience them as burdensome or distressing.

*“Maybe it sounds harsh, but you get used to some things. I think they are part of the job we choose for” (participant #3)*

*“I experienced multiple critical incidents the past two weeks… It takes more for me to get it out of my head, I am dealing with it, it is going through my head.” Interviewer: So, the consequences weigh heavier if the critical incidents occur in a shorter period of time? Participant: “Yes, it sure does” (participant #20)*

*“I do not experience any long term effects of these consequences” (participant #15)*

*“How I cope with a critical incident strongly depends on how I feel, if I have eaten well, if I slept well, if I have any other problems in my personal life… these are all factors that influence my coping” (participant #1)*

The physical consequences mentioned were an increase of heart rate and the feeling of an adrenaline rush before, during and after a critical incident, short fatigue after a critical incident, difficulties with falling asleep, and dreaming of the incident. The psychological consequences experienced were feeling less happy or sadness or irritable, feelings of being placed in an unreal situation, and an increase in appreciation of one’s own life. Reliving the incident was mentioned by several participants. The reliving could happen spontaneously or could be triggered by a question, visiting the location of the incident or by seeing the incident on (social) media. Some starting ambulance care professionals mentioned that when they are confronted with an experienced critical incident through (social) media, they relived their cases up to a year after the incident. Social consequences were taking a critical incident back home after work, and social withdrawal.

*“If I have experienced a critical incident, I can dream about it…But I know it I just my way to process the incident. However, these dreams can be very realistic: I relive the incident, transfer the incident to another setting or it focusses on my own acting ” (participant #18)*

*“Sometimes I feel sad, or I feel the pain for the family” (participant #15)*

*“Sometimes I feel irritated or I feel less happy, but these feelings always vanish over time” (Participant #6)*

*“Sometimes I relive the incident as I read or see in in the media (newspaper, tv), other times it just pops up in your mind” (participant #22)*

### Coping

To cope with critical incidents and the experienced consequences, starting ambulance care professionals apply different strategies during different phases of the ambulance care process: pre-arrival during the call/on the way to the event, on-scene, and afterwards.

All participants reported that on the way to the event they primarily focus on their medical performance to get grip and control, even if they perceived the incident as critical based on the information of the ambulance dispatch center. They try to make an mental imagination of what they might find on-scene, use their pre-arrival preparation, re-read their protocols and discuss their action plan with the ambulance driver. On-scene, the starting ambulance care professionals continue with their focus on medical performance and use their ABCDE-methods and protocols. Also, in this phase all participants depersonify the patient into a medical case.

*“If I judge an incident as critical based on the dispatch information, I discuss this with the ambulance driver and check my protocols to prepare myself…I stay away from my emotions and feelings” (participant #20)*

After the critical incident, all participants searched for support and distinguished workplace support from personal support. As for workplace support, all participants described how they shared their feelings and thoughts about the critical incident with their nearest on-scene colleague: the ambulance driver. Also, they searched for support with other ambulance care professionals, most of the time at the coffee table, and other times by actively contacting colleagues they trust. Participants mentioned that searching for workplace support has two goals: sharing their feelings and searching for confirmation on their performance by analyzing the case and reassure themselves. They also mentioned that workplace culture is essential to share their feelings regarding the critical incident.

*“I feel like I want to talk about it, especially with my colleagues. They understand me..” (participant #4)*

*“Afterwards, I talk with my colleagues about the incident. I ask them “I approached the case this way, how would you handle it?” (participant #9)*

Within their search for personal support, participants talked with family and friends and made a distinction between persons in their personal life with and without medical background. Participants perceived persons with a medical background as peers and share the content of the case and their feelings with them. In contrast, participants used persons without a medical background primarily to inform them and share their feelings.

*“It’s nice to talk about the incident with friends who weren’t there…They ask different questions than my colleagues” (participant #13)*

*“My girlfriend works in healthcare, she also experiences critical incidents. It helps that we can talk about it we relate to each other and watch over each other” (participant #11)*

Most of the participants mentioned they strived for confirmation if they medically handled the case correct. Confirmation was searched for by discussing the case with colleagues from ambulance care and the helicopter emergency medical service, by contacting the emergency department the patient was transported to, and by searching in literature or guidelines. Striving for confirmation had two goals: reassurance and input for their own professional development.

*“I really need confirmation, from my driver, from my other colleagues I worked with. Did we see the same things? Did we feel the same things? Did we do it well?.. It reassures me..” (participant #12)*

*“I contacted the physician from the emergency department to ask if I acted correctly and if I could have done any other things for this child. Just for my own learning process, but also to reassure myself of course” (participant #8)*

Most of the participants described how they seeked distraction directly after a critical incident. Distraction was sought in many different ways, ranging from seeing friends, walking with the dog, to watching a movie, making or listening to music or going to the gym.

### Training and guidance

#### Training during bachelor of health educational program

The majority of participants don’t actively remember they received education about coping with critical incidents during their bachelor of health educational program. However, few mentioned theoretical lectures about critical incidents, and that there was a support team of lecturers present which they could consult in case of an critical incident. Most participants did mention that during the 3rd and 4th year internships there were supervision sessions where they talked about critical incidents. However, the participants experienced these sessions as primarily focusing on the medical aspects of the case, and less about their feelings and coping. Participants mentioned that their lecturer could easily be approached when the student wanted to talk about a critical incident. Most starting ambulance care professionals mentioned that during the 3rd and 4th year internships they learnt the most about coping with critical incidents. Their workplace preceptor taught them that critical incidents are part of the job, that social support from colleagues and their next of kin is important, and that it is important to talk about their feelings.

*“During my initial educational program they did not spent much time on critical incidents. But that is something I needed to gain insight in what is normal and what is not” (participant #9)*

#### Training at emergency medical service

During and after the traineeship, the workplace preceptor has a crucial role for starting ambulance care professionals to learn them how to cope with critical incidents. They described the workplace preceptor as confidential counselor and someone they trust to share their feelings.

*“I had two very good preceptors. We shared our stories and experiences. We experienced it together, that helps when you have to cope with it” (participant #17)*

All starting ambulance care professionals mentioned a Critical Incident Debriefing team (CID-team). This CID-team can be activated by the dispatch center based on the call and predefined criteria or the professional. Some use this CID-team to cope with the critical incident. Others mentioned that their EMS provided the option to fill in an online form when they encountered a critical incident. One followed additional training initiated by the EMS, on how to cope with critical incidents.

## Discussion

The aim of this study was to gain insight in (a) what starting ambulance care professionals describe as critical incidents, (b) how they experience these critical incidents and their consequences, (c) how they cope with these incidents, and (d) how they are trained and guided to cope with these incidents.

From our results three key-themes emerged that make and incident critical for starting ambulance care professionals: emotional connection versus emotional detachment, feeling loss of control, and incomprehension. Emotional connection and detachment and feeling loss of control have been reported earlier in literature [3, 13–15, 19]. To our knowledge, incomprehension has not been reported earlier in literature about critical incidents amongst starting ambulance care professionals. It is possible the participants in our study, who are relatively young in age and experience, and still are at the beginning of their career, are developing their comprehensibility. The concept of comprehensibility is part of Sense of Coherence (SOC). SOC allows a person to understand why a critical incident occurred (comprehensibility), to manage it on their own/with the help of others (manageability) and to find deeper meaning (meaningfulness) [20, 21]. Recent studies indicate that higher SOC levels are associated with lower symptom severity for PTSD [22, 23]. The presence of incomprehensibility amongst the participants in our study, possibly indicates an underdevelopment of SOC. This possible underdevelopment urges the need to actively stimulate SOC development in ambulance care training and education, to prevent development of PTSD amongst starting ambulance care professionals.

Most of the described critical incidents are comparable to literature [1–3, 12–14]. Pediatric critical incidents were mentioned most often, which might be explained by the three key-themes making an incident critical being combined. A recent study focused on pediatric critical incidents and also reported on identification with the patient and the social value of children [24]. A remarkable finding of this study is that planned ambulance care can yield critical incidents, which has not been reported previously. The nature of these critical incidents is different, there is more time with patients who might be critically ill or terminal, but less time needed for assessment or interventions, and more time for personal conversation. This leads to ambulance care professionals getting to know the patient and emotionally connect to him, thus making the incident more critical. When preparing and coaching ambulance care professionals attention should be payed to these less obvious critical incidents. Also, all participants defined and described patient related critical incidents. These patient related critical incidents have a relationship with PTSD symptoms amongst emergency nurses [25]. None of the participants described workplace or organizational related critical incidents, although these were reported previously and can evoke exhaustion [12, 25, 26].

From our results it seemed that critical incidents have short-term to middle term consequences on physical, psychological and social level for starting ambulance care professionals. These were all coherent with literature on ambulance care professionals in general [2, 3, 13, 19]. Participants accepted these consequences as part of their job, and did not experience them as burdensome or distressing. Possibly, this is due to the relatively short presence of these consequences. Also, starting ambulance care professionals might not be able to recognize these possible early symptoms of mental health disorders, that are highly prevalent amongst ambulance care professionals [4–7]. Therefore, future ongoing screening for these mental health problems, and education about coping, consequences and recognizing early onset of symptoms, is recommended. Especially as evidence for interventions to improve mental health for healthcare students and starting professionals is limited [27].

Our results indicate that starting ambulance care professionals use a variety of coping strategies during different phases of the ambulance care process, which is comparable to previous studies [3]. Participants in our study used a mix of depersonification, focus on the medical task, support from colleagues and their own network, seeking confirmation, and distraction. All of these coping strategies have been reported previously [3, 13, 14, 28]. All participants stressed the importance of seeking support as coping style after exposure to a critical incident. Seeking support by colleagues who were present (the driver or another ambulance care professionals) or a colleague in the same shift has been reported as the most used strategy and as being helpful [1, 13, 28]. Despite collegial support being important and helpful, one study showed that only 44% of the ambulance care professionals experienced their peers as supportive [1]. Seeking support in their own social network has been reported, although this study also reported on ambulance care professionals feeling misunderstood by their social network [19]. When social support is lacking, ambulance care professionals face increased risks for psychological consequences [29]. To optimize support as coping style in the EMS organization, peer supporters could be implemented. A recent study investigated how ambulance care professionals perceived a peer supporter in their EMS group, as facilitator to express feelings and emotions [13]. Ambulance care professionals mainly had a positive perception, motivated by feeling certainly understood by a peer, although some suggested to combine the peer supporter with an outside psychologist as they feared being judged and lack of one’s privacy.

Our study showed that participants did not actively remember receiving education about critical incidents, especially not on the feelings these incidents evoke, this is in line with a previous study showing that only 55% of respondents had ever received any information or education about PTSD [7]. From our results emerged that the role of the workplace preceptor during education and traineeship is crucial in learning how to cope with critical incidents, and that a trustful relationship is essential. Studies underline this crucial role of the preceptor within the EMS learning environment, but also show that preceptors experience varying levels of support from universities and that preceptors are perceived as unsupportive by ambulance care trainees [30, 31]. While the EMS preceptor has a crucial role in early identification, intervention, referral and coaching, EMSs and universities should support and train preceptors, lecturers and supervisors on this topic. Besides adequate preparation during initial training and the traineeship, it is important to adequately support ambulance care professionals to cope with critical incidents and their consequences. Furthermore, it seems reasonable to gain insight in experienced critical incidents and coping among EMS students. These students are younger and might experience different critical incidents and might need different supporting interventions tailored to their specific needs. Finally, participants mentioned the CID team as possible workplace intervention after a critical incident. However, our population mentioned different kinds of critical incidents, which might not match the CID predefined criteria.

### Strengths and limitations

Strengths of the current study are the high participation rate of the approached total population of starting ambulance care professionals, the in-depth method of interviewing, the achievement of data saturation, the peer review, the member check, and a topic list being based on relevant literature. Also, this is the first study to address the bachelor of health, and gained insight in a relatively new group of ambulance care professionals. However, this novelty also limits measurement of long-lasting effects of critical incidents. Other limitations are the specific Dutch setting and the specific educational route of the participants, which might limit applicability and transferability of the results.

## Conclusion

This study shows that emotional connection, feeling loss of control, and incomprehension are key-themes that make an incident critical for starting ambulance care professionals, and that critical incidents occur during high urgency as well as planned ambulance care. These critical incidents evoked short and middle term physical, psychological and emotional consequences, although the ambulance care professionals perceived these consequences as part of their job. To cope with these critical incidents, starting ambulance care professionals use a variety of coping strategies during different phases of the ambulance care process, where support was marked as most important. Some starting ambulance care professionals remember having education and training about critical incidents, but the workplace preceptor had the most crucial role in helping starting ambulance care professionals to cope with critical incidents. These findings might serve as input for future development of educational programs, traineeships and training for ambulance care professionals.

## Data Availability

The datasets generated and/or analyzed during the current study are not publicly available due to their confident nature but a coding scheme is available from the corresponding author on reasonable request.
